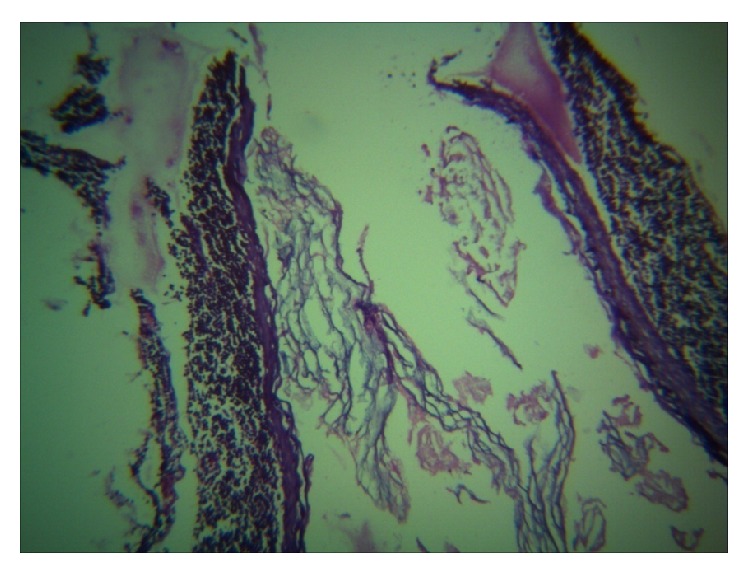# Epidermal Cyst of Parotid Gland: A Rarity and a Diagnostic Dilemma

**DOI:** 10.1155/2015/856170

**Published:** 2015-01-06

**Authors:** Anuradha Ganesan, Gautham Kumar Nandakumar

**Affiliations:** ^1^Department of Oral Medicine & Radiology, Madha Dental College & Hospital, Kundrathur, Chennai 600069, India; ^2^Department of Periodontics, Madha Dental College & Hospital, Kundrathur, Chennai 600069, India

## Abstract

Epidermal cysts are common skin lesions but they occur very rarely in the oral cavity, especially in the salivary glands. Very few cases have been reported in the literature and, here, we present one such rare case of epidermal cyst in the right parotid gland in a 62-year-old female patient.

## 1. Introduction

Epidermal/epidermoid cysts are common lesions occurring in the skin [1]. Only 1.6% occur in the oral cavity and are rare [2]. However, primary epidermal cysts of salivary glands appear to be very rare and literature search for the past 25 years revealed only very few cases in parotid gland [3] and some cases in submandibular gland [1, 4, 5]. The epidermal cyst is a benign cyst and develops out of ectodermal tissue. The several synonyms are epidermal cyst, epidermal inclusion cyst, infundibular cysts, and keratin cysts [6].

The diagnosis of an epidermal cyst in the parotid gland becomes very essential and it is a very rare entity and it could be easily mistaken for a salivary gland abscess, neoplasm, and other cysts [7]. Therefore, an excisional biopsy is necessary for a prompt diagnosis and confirmation.

## 2. Case History

A 62-year-old female patient presented to our outpatient department with a complaint of swelling on the right side of the face in front of the ear for two years. The swelling was insidious in onset and gradually progressed to reach the present size. There was no history of pain, fever, difficulty in swallowing, or any discharge from the swelling. There were no other swellings present anywhere else in the body. There was also no history of trauma or any previous surgeries reported in the facial region.

**Figure 1:**
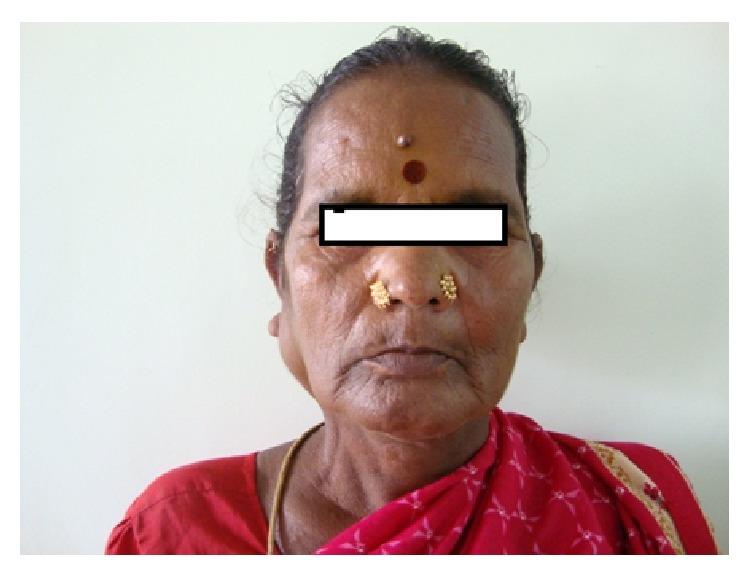


**Figure 2:**
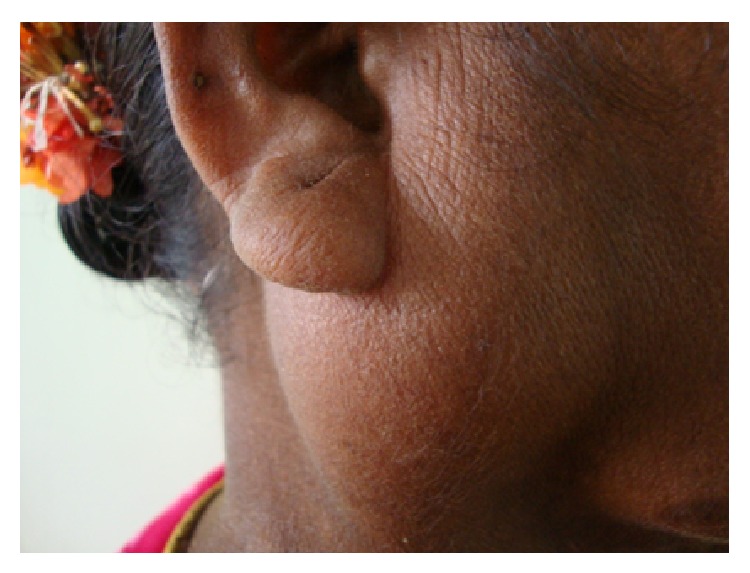


**Figure 3:**
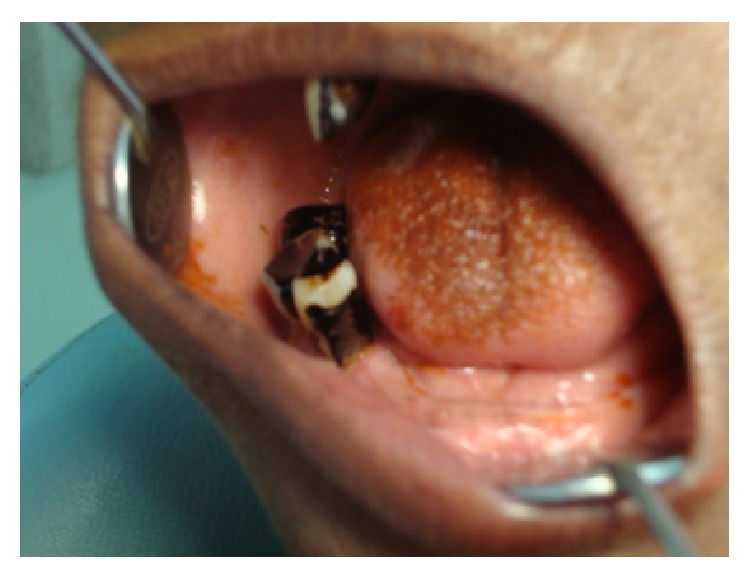


On examination, there was a localized ovoid swelling in the right preauricular region. The swelling was 6 × 8 cm in size and extended around 2 cm below the lobule of the right ear. There was no lifting of the ear lobe and the colour over the swelling was of normal skin colour with no surface discharge (Figures 1 and 2). On palpation, the swelling was soft in consistency, nontender, and nonpulsatile and was movable below the skin. Intraorally, there was no swelling present and multiple teeth were missing and mobility in tooth numbers 45, 46, and 47 was present (Figure 3).

Ultrasound was carried out and it showed hyperechoic cystic lesion in the right parotid region measuring 4.2 × 6.1 cm. There was no vascularity in the lesion and no evidence of calculi in the duct or glands. So a benign parotic cystic salivary gland lesion was given as a diagnosis.

**Figure 4:**
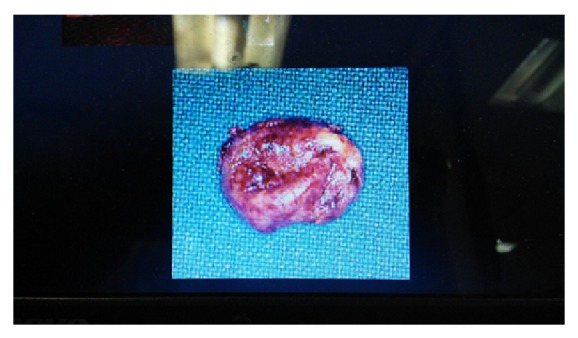


**Figure 5:**
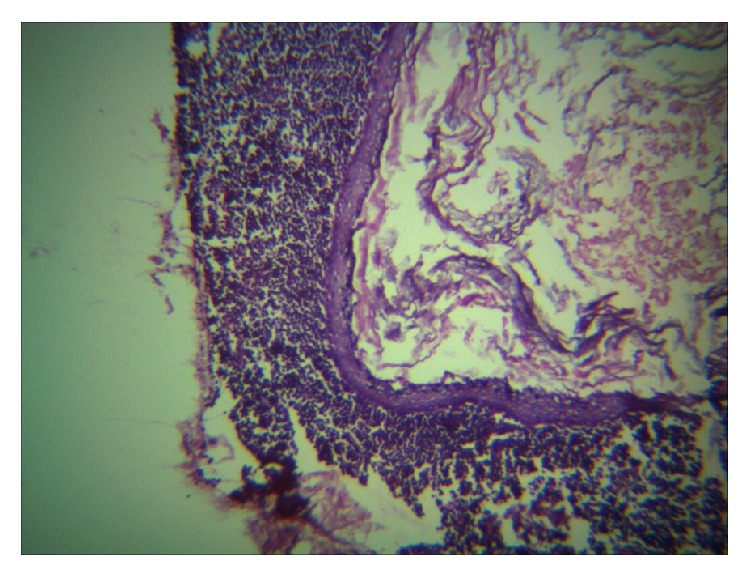


Patient underwent surgical intervention and superficial parotidectomy was carried out. The cyst was removed in toto and gross examination revealed a globular mass measuring 4.5 × 6 cm in size and cut surface yields a pultaceous material (Figure 4). Sections were made and histopathological examination revealed stratified squamous epithelium with an intraluminal laminated keratinized material confirming the diagnosis of epidermal cyst in the right parotid gland (Figures 5 and 6). Post operatively the healing was uneventful and regular follow up for a year showed no signs of recurrence.

## 3. Discussion

Epidermal cysts are common skin lesions that consist of epithelial lined cavities which are filled with viscous or semisolid epithelial degradation products [8]. Epidermal cysts of the oral cavity are a very rare entity and only 1.6–6.9% of all epidermal cysts are thought to be located in the oral cavity [9]. Epidermal cysts usually occur secondary to obstruction while dermoid cysts arise from developmental epithelial remnants or they are secondary to traumatic implantation of epithelial fragments [10].

Epidermal cyst of parotid gland is a very rare benign cystic lesion and is seen in young to middle age adults [6]. The exact histogenesis of salivary epidermal cyst is uncertain, but it may have arisen from developmental branchial pouch analogue epithelium which can occur in salivary gland [11] or could be due to obstruction in salivary duct within the substance of the gland leading to epithelial lining cavity filled with viscous semisolid epithelial degradation product [3] as seen in our case. The cysts clinically are painless swellings without any attachment to the overlying skin or involvement of facial nerve [6]. If the cyst stays for longer time, it might get infected forming sinus or fistulas [3].

The different causes of swelling in the parotid region may include branchial cleft cyst which is “congenital”, or may be “acquired” due to inflammation, obstruction, neoplasm, calculi and trauma [6]. Also if it occurs in the submandibular region, it can be mistaken for salivary gland abscess, neoplasm, tuberculous lymphadenitis, metastatic node, or any cyst [1, 12]. The diagnosis can be proven by various investigations like FNAC, ultrasound, and CT [2, 13]. The diagnosis of the cystic lesion is challenging due to difficulty in determining the benign or malignant processes. Malignant lesions are frequently suspected when there is a rapid enlargement with associated lymphadenopathy or facial nerve paralysis [6, 14]. The treatment is surgical excision of the cyst. Care should be taken not to rupture the cyst which can lead to postoperative inflammation and also to preserve the vital structures during surgery [3].

Histopathological examination of the cyst is required for confirmation of diagnosis. Histologically, epidermal cyst has stratified squamous epithelial lining and is usually filled with cheesy material or keratin. But a dermoid or epidermoid cyst contains skin adnexa or other epidermal structures like sebaceous gland or hair follicle. Implantation dermoid is not derived from epidermal appendages and may contain foreign body [9] even though it appears very similar to epidermoid cyst. Recurrence is very rare.

## 4. Conclusion

Epidermal cysts of the parotid gland origin are extremely rare and a diagnostic challenge, but still, epidermal cysts should be considered as a differential diagnosis in cases of painless long standing enlargement of parotid gland which is soft in consistency.

## Figures and Tables

**Figure 6:**